# Clinico-Hematological Profile of Children and Adolescents With Chronic Myeloid Leukemia: A Study at a Tertiary Care Institute in India

**DOI:** 10.7759/cureus.82673

**Published:** 2025-04-21

**Authors:** Kaushal Kumar, Rawi Agrawal, Satish Kumar

**Affiliations:** 1 Department of Hematology, Indira Gandhi Institute of Medical Sciences, Patna, IND

**Keywords:** abelson leukemia virus, breakpoint cluster region, children and adolescents, chronic myeloid leukemia, pediatrics, tyrosine kinase inhibitors

## Abstract

Background

Chronic myeloid leukemia (CML) rarely occurs in the first few decades of life, accounting for 2% to 3% of leukemias in children and adolescents. Children and adolescents with CML tend to present with more aggressive features. Because of the substantial shift in CML treatment brought about by the development of tyrosine kinase inhibitors (TKIs) in the last 20 years, adult patients now have about the same life expectancy as the age-matched healthy population. The study evaluated the clinico-hematological profile of children presenting with CML. Also, the response to the treatment provided was analyzed.

Materials and methods

It is a retrospective-observational study conducted in the Department of Hematology, Indira Gandhi Institute of Medical Sciences (IGIMS), Patna, Bihar, India. The Institutional Ethics Committee (IEC), IGIMS, Patna, Bihar, India, granted ethical approval under letter 1370/IEC/IGIMS/2024, dated 20 March 2024.

Results

Out of 180 cases detected from records of the previous six years, i.e., from 2018 to 2023, only 20 records of CML were found in children and adolescents. Among them, 17 (85%) patients had splenomegaly, 16 (80%) had anemia, 10 (50%) had hepatomegaly, and two (10%) had fever.

Conclusion

The study concluded that the presenting features of CML in children and adolescents were similar to those shown in other studies. WBC counts were higher in children and adolescents compared to the adult population. Also, the long-term outcomes of TKI treatment and the late effects of life-long TKI treatment need to be defined.

## Introduction

About 2%-3% of leukemias in children and adolescents are chronic myeloid leukemia (CML) [1,2]. According to epidemiologic data, the incidence rises with age and ranges from 0.6 to 1.2 per million children annually. In infancy, CML is extremely uncommon, occurring in 0.7 out of every million children aged one to 14 and 1.2 out of every million teenagers annually [1]. The adult population is the main target of CML, which is extremely uncommon among children and adolescents [3].

It has been noted that CML of children and adolescents differs from CML of adults [1,4]. Higher WBC counts, larger spleens relative to body size, and a higher incidence of advanced stages at diagnosis are some of the more aggressive characteristics that children and adolescents with CML typically exhibit [5].

CML is seen to be developed when genes present on chromosomes 9 and 22 are reciprocally translocated to form the Breakpoint Cluster Region-ABelson leukemia virus-1 (BCR-ABL1) fusion gene, which further elevates tyrosine kinase activity and stimulates the proliferation and differentiation of myeloid [6,7].

Because of the substantial shift in CML treatment brought about by the development of tyrosine kinase inhibitors (TKIs) in the last 20 years, adult patients now have about the same life expectancy as the age-matched healthy population [8,9]. Outcomes in children have also improved with imatinib treatment [5,10,11]. However, the recent approval of dasatinib and nilotinib as first-line treatments for children was found to be more grateful toward the treatment of CML [12].

None of the numerous validated adult scoring systems for evaluating therapy response have been particularly tested in the juvenile population [13]. The study has been conducted to evaluate the clinico-hematological profile of children presenting with CML. Also, the response to the treatment provided was analyzed.

## Materials and methods

Study design

It is a retrospective-observational study. The study was conducted in the Department of Hematology, Indira Gandhi Institute of Medical Sciences (IGIMS), Patna, Bihar, India. The data from the previous six years was taken, i.e., from 2018 to 2023.

Study population

A total of 180 patients were enrolled in the study. The inclusion criteria were patients under 18 years of age who presented with relevant clinical features, such as abdominal pain and weakness, along with hematological findings suggestive of CML, including anemia, Ph chromosome positivity, and findings from bone marrow examination. Additionally, patients in blast crisis of CML presenting as acute leukemia were included. The exclusion criteria included patients who had received prior chemotherapy or radiotherapy, Ph chromosome-negative CML patients (as only Ph chromosome-positive patients were included), and previously diagnosed and treated CML patients, as these factors could affect the clinico-hematological profile.

Data collection

The data were collected from the records, including age, symptoms, clinical findings, and stages of CML in children and adolescents. Additionally, laboratory findings and responses to TKI therapy were recorded. Response to TKI receptors was also documented.

Statistical analysis

Data was entered in Microsoft Excel (Microsoft Corporation, Redmond, Washington). Then, Statistical Package for the Social Sciences (SPSS) version 24 (IBM Corp., Armonk, NY, USA) was used for further statistical analysis. Data were presented as either n (%) or mean±SD. An Independent t-test was used to obtain the p-value. A P-value less than 0.05 was considered significant.

Ethical clearance

The Institutional Ethics Committee (IEC), IGIMS, Patna, Bihar, India, granted ethical approval under letter number 1370/IEC/IGIMS/2024, dated 20 March 2024.

## Results

Out of all 180 cases, only 20 records of CML were found in children and adolescents. Of those 20 patients, five (2.78%) participants were in the age group of <13 years, while 15 (8.3%) participants were in the age group of 13-18 years. Table 1 represents the number of cases of CML detected in participants.

**Table 1:** Number of CML cases detected in participants Data have been presented as n (%). CML, chronic myeloid leukemia

Year	Total number of cases	Age (<13 years)	Age (13-18 years)
2018	20 (11.1%)	00 (0%)	01 (6.6%)
2019	21 (11.6%)	00 (0%)	06 (40%)
2020	25 (13.8%)	00 (0%)	00 (0%)
2021	06 (3.3%)	00 (0%)	01 (6.6%)
2022	42 (23.3%)	02 (40%)	02 (13.3%)
2023	66 (36.6%)	03 (60%)	05 (33.3%)
Overall cases	180	05	15

Among 20 cases of CML in children and adolescents, 17 (85%) were in the chronic phase, two (10%) in the accelerated phase, and one (5%) in the blast phase. Table 2 represents the phases of CML detected in participants.

**Table 2:** Phases of CML detected in participants Data were presented as n (%). CML, chronic myeloid leukemia

Year	Chronic phase	Accelerated phase	Blast phase
2018	01 (5%)	00 (0%)	00 (0%)
2019	04 (20%)	00 (0%)	01 (5%)
2020	00 (0%)	00 (0%)	00 (0%)
2021	01 (5%)	00 (0%)	00 (0%)
2022	03 (15%)	02 (10%)	00 (0%)
2023	08 (40%)	00 (0%)	00 (0%)

Clinical findings along with symptoms in children and adolescents with CML have been elaborated in Table 3. Among clinical findings, 17 (85%) patients had splenomegaly, 16 (80%) had anemia, 10 (50%) had hepatomegaly and, two (10%) had fever. Abdominal fullness was observed in 16 (80%) of patients, abdominal pain in 12 (60%), and weakness in 11 (55%).

**Table 3:** Clinical findings in children with CML Data were presented as n (%). CML, chronic myeloid leukemia

Clinical findings	Number of patients
Splenomegaly	17 (85%)
Anemia	16 (80%)
Hepatomegaly	10 (50%)
Hypermetabolic state; fever	02 (10%)
Symptoms	
Abdominal fullness	16 (80%)
Abdominal pain	12 (60%)
Weakness	11 (55%)

Table 4 depicts the laboratory findings observed among participants. The laboratory findings were compared between the pediatric and adult populations. There were no statistically significant differences observed between the groups of pediatric and adult populations.

**Table 4:** Laboratory findings among participants Data were presented as mean±SD. Independent t-test was used to evaluate the p-value. P-value was considered significant at <0.05.

Laboratory findings	Reference ranges	Pediatric population (n=20)	Adult population (n=160)	T-value	P-value
Hemoglobin (g/dL)	12-16 g/dL	8.1±1.2	8.7±1.4	1.8	0.06
WBC (×10^3^/µL)	4.5-11 ×10^3^/µL	3.02±0.98	2.95±0.94	-0.313	0.75
Platelets (×10^3^/µL)	150-450 ×10^3^/µL	443.2±128.6	412.8±114.8	-1.1	0.27
Lactate dehydrogenase (IU/L)	125-220 IU/L	1023.3±287.8	992.1±103.2	-0.97	0.33
Uric acid (mg/dL)	3.5-7.2 mg/dL	4.9±2.1	5.2±1.9	0.65	0.51
Alkaline phosphatase (IU/L)	44-147 IU/L	143.6±68.2	153.6±59.2	0.7	0.48

Among the pediatric population, very few children were presented at different follow-ups for recording the response to TKI therapy. Overall, most of the participants were present at the last follow-up. Table 5 represents the response to TKI therapy among participants.

**Table 5:** Response to TKIs therapy Data were presented as n (%). BCR-ABL1, Breakpoint Cluster Region-ABelson leukemia virus-1; TKI, tyrosine kinase inhibitors

BCR-ABL1	At 3 months (n=12)	At 6 months (n=14)	At 12 months (n=15)	Last follow-up (n=18)
>10%	05 (41.6%)	02 (14.2%)	04 (26.6%)	06 (33.3%)
>1%-10%	07 (58.3%)	05 (35.7%)	02 (13.3%)	04 (22.2%)
>0.1%-1%	00 (0%)	03 (21.4%)	03 (20%)	05 (27.7%)
≤0.1%	00 (0%)	04 (28.5%)	06 (40%)	03 (16.7%)

## Discussion

This study has been conducted to evaluate clinico-hematological findings among children and adolescents with CML. Among all the data of 180 patients, 20 records were found of children and adolescents. Of the 20 cases, 15 (75%) patients were between 13 and 18 years of age. According to a retrospective study conducted in 2023 by Jetly D et al. on 44 newly diagnosed BCR-ABL1-positive patients, the majority, 16 patients (36.4%), were between 11 and 15 years of age [14].

The chronic phase of CML accounted for the majority of the records discovered, followed by accelerated and blast phases. Our outcomes were found to be in concordance with a study conducted by Jetly D et al., which also reported that the chronic phase was found prominently among the pediatric population [14].

Splenomegaly and anemia were present predominantly in 17 (85%) and 16 (80%) patients, respectively. A similar outcome was reported in the study by Jetly D et al. in 2023, which stated that among 44 cases of CML, splenomegaly was a prominent finding in 42 (95.5%) patients [14]. Another study by Kumar K et al. also reported that out of 58 cases, splenomegaly was observed in the majority of patients, i.e., in 51 (87.9%) patients as a sign of CML, and anemia was seen in 57 (98.27%) patients [15].

Hepatomegaly was observed in 10 (50%) patients in our study. Studies like Raut L et al. in 2013 and Chandra D et al. in 2018 reported that among the pediatric population, hepatomegaly was present in 14% and 35% of children, respectively [16,17].

Symptoms such as abdominal fullness and abdominal pain were observed in 16 (80%) and 12 (60%) patients, respectively, in our study. These findings were similar to the findings observed in respective studies conducted by Millot F et al. in 2011 and Chandra D et al. in 2018, as abdominal pain was observed in 37% and 37.3% of patients, respectively [5,17].

In the study, the mean WBC count was 302.5×10^3^/µL. The mean LDH and ALP were 1023.3 IU/L and 143.6 IU/L, respectively, similar to the study by Jetly D et al., which reported the levels of LDH and ALP as 1124.1 and 150.5 IU/L, respectively, along with levels of WBC as 311.3±189.9×10^3^/µL among children [14]. In contrast to our investigation, Hasan K et al. study had somewhat lower average ALP and LDH levels, at 122.6 U/L and 813.6 IU/L, respectively [18].

WBCs, platelets, and LDH were observed to be higher in the pediatric population compared to the adult population in the study. When we compared all the laboratory findings between children and adults, none of the results showed any significant differences, as all the p-values were above 0.05.

The most effective treatment for both adult and pediatric CML cases is a TKI.

In our study, the response to TKI therapy was also recorded. It was observed that the highest number of responses, seen in 18 patients, occurred at the last follow-up.

The main limitation of this study is that it may not represent the most advanced approaches to treating or managing CML. However, it reflects the real-world scenario of patient care in a government-run tertiary care facility in a developing nation. Additionally, inconsistencies in patient follow-up posed a significant challenge in accurately tracking treatment responses.

## Conclusions

The study concluded that the presenting features of CML in children and adolescents were similar to those shown in other studies. WBC counts were higher in children and adolescents compared to the adult population. It is also necessary to define the late impacts of lifelong TKI treatment as well as the long-term results of TKI treatment. Due to advancements in the management of adult CML patients over the last 20 years, TKI medication may now be stopped under specific circumstances. Due to the rarity of pediatric CML, more pediatric data collection is required, which can only be accomplished through international cooperation.

## References

[1] Hijiya N, Schultz KR, Metzler M, Millot F, Suttorp M (2016). Pediatric chronic myeloid leukemia is a unique disease that requires a different approach. Blood.

[2] Hijiya N, Millot F, Suttorp M (2015). Chronic myeloid leukemia in children: clinical findings, management, and unanswered questions. Pediatr Clin North Am.

[3] Ries LAG, Smith MA, Gurney JG (1999). Cancer Incidence and Survival Among Children and Adolescents: United States Seer Program, 1975-1995. National Cancer Institute.

[4] Castagnetti F, Gugliotta G, Baccarani M (2015). Differences among young adults, adults and elderly chronic myeloid leukemia patients. Ann Oncol.

[5] Millot F, Baruchel A, Guilhot J (2011). Imatinib is effective in children with previously untreated chronic myelogenous leukemia in early chronic phase: results of the French national phase IV trial. J Clin Oncol.

[6] Quintás-Cardama A, Cortes J (2009). Molecular biology of bcr-abl1-positive chronic myeloid leukemia. Blood.

[7] Sawyers CL (1999). Chronic myeloid leukemia. N Engl J Med.

[8] Bower H, Björkholm M, Dickman PW, Höglund M, Lambert PC, Andersson TM (2016). Life expectancy of patients with chronic myeloid leukemia approaches the life expectancy of the general population. J Clin Oncol.

[9] Hijiya N, Suttorp M (2019). How I treat chronic myeloid leukemia in children and adolescents. Blood.

[10] Suttorp M, Schulze P, Glauche I (2018). Front-line imatinib treatment in children and adolescents with chronic myeloid leukemia: results from a phase III trial. Leukemia.

[11] Champagne MA, Fu CH, Chang M (2011). Higher dose imatinib for children with de novo chronic phase chronic myelogenous leukemia: a report from the Children's Oncology Group. Pediatr Blood Cancer.

[12] Gore L, Kearns PR, de Martino ML (2018). Dasatinib in pediatric patients with chronic myeloid leukemia in chronic phase: results from a Phase II trial. J Clin Oncol.

[13] Ganta RR, Nasaka S, Linga VG, Gundeti S, Maddali LS, Digumarti RR (2017). Effectiveness of three prognostic scoring systems in predicting the response and outcome in pediatric chronic myeloid leukemia chronic phase on frontline imatinib. Indian J Med Paediatr Oncol.

[14] Vanik SA, Jetly D, Parikh B, Dhandapani K, Bezbaruah R (2023). Pediatric and adolescent chronic myeloid leukemia: a follow-up study in western India. Indian J Pathol Microbiol.

[15] Kumar K, Agrawal R, Kumar S, Iffat Jamal S, Ranjan A, Choudhary VN (2022). Clinico - hematological profile and bone marrow correlation of chronic myeloid leukemia: a multiparameter study in a tertiary care hospital. Eur J Mol Clin Med.

[16] Raut L, Bohara VV, Ray SS, Chakrabarti P, Chaudhuri U (2013). Chronic myeloid leukemia in children and adolescents: a single center experience from Eastern India. South Asian J Cancer.

[17] Chandra D, Singh J, Deka R (2018). Biology of chronic myelogenous leukemia in childhood and young adolescents: an Indian perspective. Indian J Med Paediatr Oncol.

[18] Hasan K (2018). Evaluation of chronic myeloid leukemia patients and their molecular responses to tyrosine kinase inhibitors in Erbil city, Iraq. Iraqi J Hematol.

